# Quercetin inhibits Cr(VI)-induced malignant cell transformation by targeting miR-21-PDCD4 signaling pathway

**DOI:** 10.18632/oncotarget.10130

**Published:** 2016-06-17

**Authors:** Poyil Pratheeshkumar, Young-Ok Son, Sasidharan Padmaja Divya, Lei Wang, Lilia Turcios, Ram Vinod Roy, John Andrew Hitron, Donghern Kim, Jin Dai, Padmaja Asha, Zhuo Zhang, Xianglin Shi

**Affiliations:** ^1^ Center for Research on Environmental Disease, University of Kentucky, Lexington, KY, USA; ^2^ Department of Toxicology and Cancer Biology, University of Kentucky, Lexington, KY, USA; ^3^ Department of Surgery, University of Kentucky, College of Medicine, Lexington, KY, USA; ^4^ National Centre for Aquatic Animal Health, Cochin University of Science and Technology, Cochin, India

**Keywords:** hexavalent chromium, quercetin, ROS, malignant cell transformation, miR-21-PDCD4 signaling

## Abstract

Hexavalent chromium [Cr(VI)] is an important human carcinogen associated with pulmonary diseases and lung cancer. Inhibition of Cr(VI)-induced carcinogenesis by a dietary antioxidant is a novel approach. Quercetin is one of the most abundant dietary flavonoids widely present in many fruits and vegetables, possesses potent antioxidant and anticancer properties. MicroRNA-21 (miR-21) is a key oncomiR significantly elevated in the majority of human cancers that exerts its oncogenic activity by targeting the tumor suppressor gene programmed cell death 4 (PDCD4). The present study examined the effect of quercetin on the inhibition of Cr(VI)-induced malignant cell transformation and the role of miR-21-PDCD4 signaling involved. Our results showed that quercetin decreased ROS generation induced by Cr(VI) exposure in BEAS-2B cells. Chronic Cr(VI) exposure induced malignant cell transformation, increased miR-21 expression and caused inhibition of PDCD4, which were significantly inhibited by the treatment of quercetin in a dose dependent manner. Nude mice injected with BEAS-2B cells chronically exposed to Cr(VI) in the presence of quercetin showed reduced tumor incidence compared to Cr(VI) alone treated group. Stable knockdown of miR-21 and overexpression of PDCD4 or catalase in BEAS-2B cells suppressed Cr(VI)-induced malignant transformation and tumorigenesis. Taken together, these results demonstrate that quercetin is able to protect BEAS-2B cells from Cr(VI)-induced carcinogenesis by targeting miR-21-PDCD4 signaling.

## INTRODUCTION

Lung cancer is globally responsible for 1.4 million deaths annually and is the leading cause of cancer-related deaths in both women and men [1]. Hexavalent chromium compounds [Cr(VI) compounds], widely used in industry, have been classified as human carcinogens by the International Agency for Research on Cancer (IARC) of the World Health Organization (WHO) based on the increased risk of lung cancer [2]. Excess production of reactive oxygen species (ROS) suggested to play a major role in Cr(VI)-induced carcinogenesis [3]. MicroRNAs (miRNAs) are a class of endogenous small (18-25 nucleotides long) non-coding RNAs that regulate target gene expression through binding to the 3′-untranslated regions (3′UTRs) of target mRNAs at the translational level [4]. It has previously been reported that ≤30% of human genes are regulated by miRNAs [5]. Among various miRNAs, miR-21 described as a key oncomiR [6], was found to be overexpressed in different types of human cancers and implicated in various aspects of carcinogenesis, apoptosis resistance, cell proliferation, tumor progression, invasion and chemoresistance [7–14]. Accumulating evidence suggests that NADPH oxidase-derived ROS is essential for the expression and function of miR-21 [15, 16].

**Figure 1:**
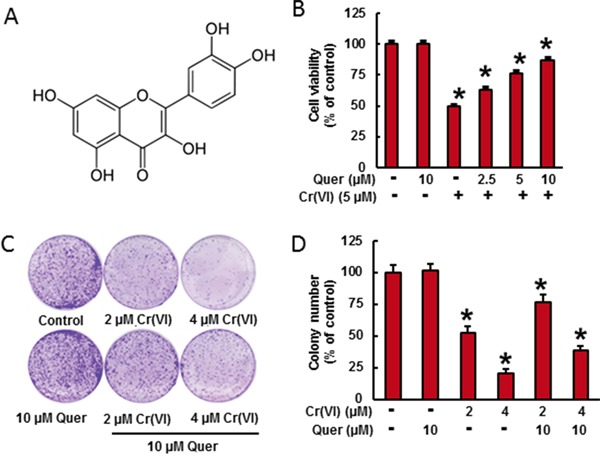
Quercetin inhibits Cr(VI)-induced cytotoxicity **A.** Chemical structure of quercetin. **B.** BEAS-2B cells were treated with Cr(VI) (5 μM) for 24 h in the presence of quercetin (0, 2.5, 5, 10 μM). Cell viability was determined by MTT assay. **C-D.** BEAS-2B cells were treated with 2 μM or 4 μM Cr(VI) with or without 10 μM quercetin for 48 h, reseeded and cultured in drug free medium for an additional 7 days and stained with crystal violet. Colony numbers in the entire dish were counted. Data presented in the bar graphs are the mean ± SD of three independent experiments. *indicates a statistically significant difference from control cells with p<0.05.

Programmed cell death 4 (PDCD4) is a novel tumor suppressor gene involved in inhibiting neoplastic transformation, and invasion, and tumor progression [4, 17–19]. Substantial data indicate that PDCD4 is consistently downregulated in human cancers and cancer cell lines [19–22]. PDCD4 is a direct target of miR-21 (binds to 3′ UTR region of mRNA), where it post-transcriptionally down regulates its expression [23–25]. Notably, decreased PDCD4 expression has been reported to be inversely correlated with miR-21 level in different tumors [22–28]. Phytochemicals and dietary compounds have been used for the treatment of cancer throughout history due to their safety, low toxicity, and general availability [29]. Quercetin (3,3′,4′,5,7-pentahydroxyflavone, Figure 1A) is the most abundant dietary flavonoid found in a variety of plant-based foods such as red onions, apples, tea, broccoli, capers, lovage, parsley, red grapes and a number of berries [30]. Chemopreventive properties of quercetin have been demonstrated in various animal models [31–33]. Previously we have shown that quercetin could inhibit angiogenesis mediated human prostate tumor growth by targeting VEGFR-2 regulated AKT/mTOR/P70S6K signaling pathways [34].

In the present study, we investigated the protective effect of quercetin on Cr(VI)-induced malignant transformation of human bronchial epithelial cells and with a focus on the key molecular events involved. We found that quercetin inhibited Cr(VI)-induced miR-21 elevation and PDCD4 reduction thereby inhibited malignant transformation of BEAS-2B cells. The treatment of quercetin significantly inhibited the Cr(VI)-induced ROS generation, where ROS is critical for miR-21 elevation and PDCD4 reduction in Cr(VI)-induced malignant transformation. In addition, quercetin also suppressed the Cr(VI)-induced tumorigenicity of BEAS-2B cells in Nude mice. These results suggest that quercetin protects BEAS-2B cells from Cr(VI)-induced malignant transformation by targeting miR-21 and PDCD4 signaling pathways.

## RESULTS

### Quercetin inhibits Cr(VI)-induced cell viability loss in culture

Cr(VI)-induced cell viability loss in culture was determined by MTT assay (Figure 1B). In BEAS-2B cells, acute treatment of Cr(VI) (5μM) caused a drastic decrease (49%) in cell viability and treatment of quercetin (5 and 10 μM) significantly ameliorates the Cr(VI)-induced cell viability loss in a dose dependent manner. The above result was further confirmed by clonogenic assay (Figure 1C). Cr(VI) at 2 and 4 μM significantly decreased the colony number, 47 % and 79 % respectively, whereas treatment with quercetin (10 μM) inhibited the adverse effect of Cr(VI) by increasing colony number (Figure 1D).

### Quercetin inhibits Cr(VI)-induced ROS generation

**Figure 2:**
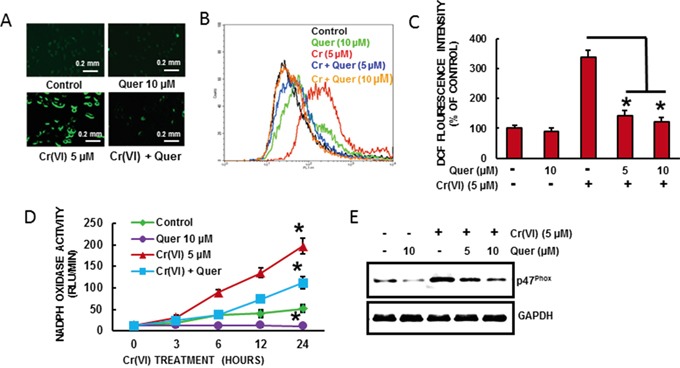
Quercetin inhibits Cr(VI)-induced ROS generation BEAS-2B cells were exposed to Cr(VI) (0 or 5 μM) with or without quercetin (0, 5, 10 μM) for 12 h and then were labeled with **A-C.** DCFDA (10 μM). Images were taken with fluorescence microscopy and fluorescent intensity was determined by flow cytometry. **D.** NOX activity was measured by lucigenin chemiluminescence assay with Cr(VI) (0 or 5 μM) in the presence of quercetin (10 μM) for indicated times. **E.** Quercetin inhibits Cr(VI)-induced protein levels of NOX subunit, p47^phox^. Data presented in the bar graphs are the mean ± SD of three independent experiments. *indicates a statistically significant difference from control cells with p<0.05.

We need to demonstrate the effect of quercetin on Cr(VI)-induced ROS generation in BEAS-2B cells. Cr(VI)-induced ROS production was quantified by flow cytometry using the fluorescent probe DCFDA. Cr(VI) exposure dramatically stimulated H_2_O_2_ generation as indicated by an increase of DCFDA (Figure 2A-2C) fluorescence intensity respectively in BEAS-2B cells compared to untreated control cells. Pretreatment with quercetin (5 and 10 μM) significantly decreased the Cr(VI)-induced H_2_O_2_ generation (Figure 2A-2C). NADPH Oxidase (NOX) is an important source of Cr(VI)-induced ROS production [3]. To study the effect of quercetin on Cr(VI)-induced NADPH Oxidase, we measured NOX activity (Figure 2D). BEAS-2B cells exposed to 5 μM Cr(VI) caused a time-dependent increase in NOX activation whereas co-treatment with quercetin significantly (p<0.05) reduced the activity. Moreover we found that acute Cr(VI) treatment also increased the expression of p47phox, one of the NOX subunits (Figure 2E), whereas co-treatment with quercetin markedly suppressed the p47phox expression. Taken together, the results suggest that quercetin able to inhibit Cr(VI)-induced ROS generation and NOX activation.

### Quercetin ameliorates Cr(VI)-induced miR-21 elevation and PDCD4 reduction

**Figure 3:**
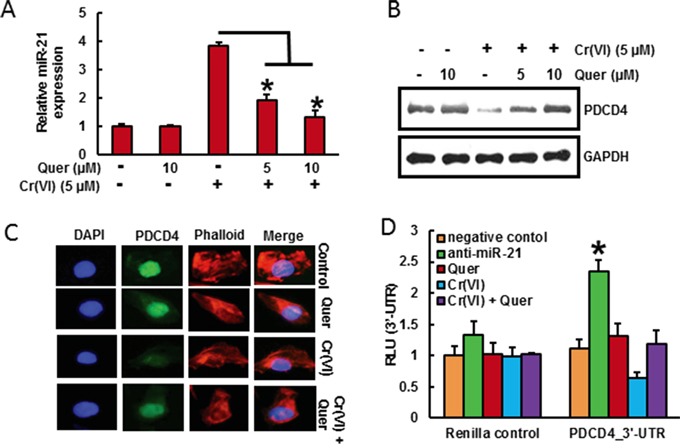
Quercetin inhibits Cr(VI)-induced miR-21 elevation and PDCD4 reduction BEAS-2B cells were treated with Cr(VI) (5 μM) for 24 h in the presence of quercetin (0, 5, 10 μM). **A.** The relative miR-21 level was determined by Taqman real-time PCR. **B.** PDCD4 protein levels after acute Cr(VI) treatment was detected by immunoblotting **C.** Representative images of fluorescence immunostaining of PDCD4 **D.** Quercetin ameliorates the Cr(VI)-induced inhibition of PDCD4 3′-UTR reporter activity. BEAS-2B cells were transfected with renilla reporter construct (pGL3-PDCD4_3′-UTR), miR-21 inhibitor (100 nM), negative control (100 nM), and pGL3-promoters and treated with 5 μM Cr(VI) for 6 h in the presence of quercetin (10 μM). Cellular lysates were subjected to a luciferase reporter assay as described in Materials and Methods. The results are expressed as relative activity (relative luminescence units (RLU)) normalized to the luciferase activity in the vector control cells without treatment. Data presented in the bar graphs are the mean ± SD of three independent experiments. *indicates a statistically significant difference from control cells with p<0.05.

It has been shown that PDCD4, a novel tumor suppressor is an important functional target of the oncogenic microRNA miR-21 [37]. As shown in Figure 3A and 3B, acute Cr(VI) treatment markedly increased miR-21 level associated with decrease in PDCD4 expression by RT-PCR and Western blot analysis respectively in BEAS-2B cells. Treatment with quercetin (5 and 10 μM) significantly (p<0.05) inhibited Cr(VI)-induced miR-21 elevation and PDCD4 reduction (Figure 3A and 3B). Similar results were observed by immunofluorescence analysis of PDCD4, where quercetin distinctly inhibited acute Cr(VI)-induced suppression of PDCD4 expression in the nucleus (Figure 3C). The treatment of Cr(VI) markedly decreased the PDCD4 3′-UTR reporter activity, whereas co-treatment with quercetin ameliorates the Cr(VI)-induced inhibition of reporter activity in BEAS-2B cells (Figure 3D). In addition, there was an upregulation in the PDCD4 3′-UTR reporter activity when miR-21 gene expression was inhibited (Figure 3D). These results demonstrate that quercetin could inhibit acute Cr(VI)-induced miR-21 elevation and associated PDCD4 reduction.

### Quercetin inhibits malignant cell transformation, miR-21 elevation and PDCD4 reduction induced by chronic Cr(VI) exposure

**Figure 4:**
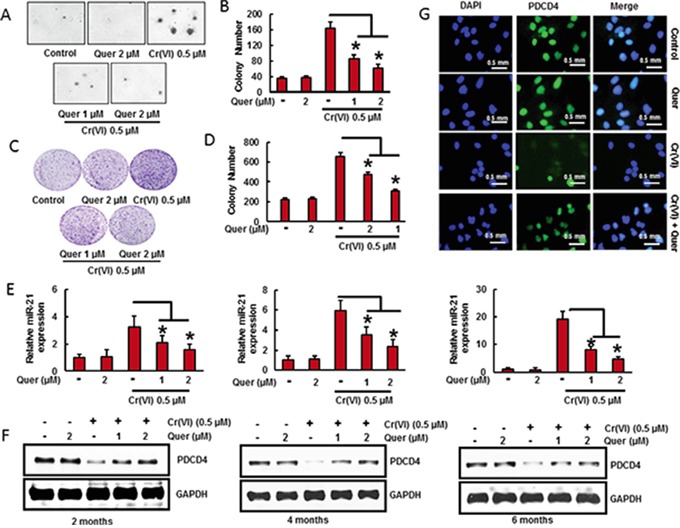
Quercetin inhibits chronic Cr(VI)-induced malignant cell transformation, miR-21 elevation and PDCD4 reduction BEAS-2B cells were maintained in a medium containing Cr(VI) (0 or 0.5 μM) with or without quercetin (1 and 2 μM) for 6 months. **A-B.** Cells were cultured in 0.35% soft agar for 5 weeks. Colony numbers in the entire dish were counted. **C-D.** Cells cultured in drug free medium for an additional 7 days and stained with crystal violet. Colony numbers in the entire dish were counted. **E.** The relative miR-21 level was determined by Taqman real-time PCR. **F.** PDCD4 protein levels after co-treatment of quercetin with Cr(VI) was detected by immunoblotting **G.** Representative images of fluorescence immunostaining of PDCD4. Data presented in the bar graphs are the mean ± SD of three independent experiments. *indicates a statistically significant difference from control cells with p<0.05.

Chronic Cr(VI) exposure induces malignant transformation in BEAS-2B cells [3]. Transformation ability was assessed by anchorage-independent growth in soft agar [38]. The chronic exposure of BEAS-2B cells with Cr(VI) (0.5 μM) for long term (6 months) induced malignant transformation of BEAS-2B cells as shown by the marked increase in size and number of colonies compared to untreated control (Figure 4A-4B). However, co-treatment of quercetin with Cr(VI) significantly (p<0.05) decreased the cell transformation and anchorage-independent growth in soft agar. In addition, quercetin also decreased the chronic Cr(VI)- induced colony formation as shown in clonogenic assay (Figure 4C-4D). We have co-treated quercetin (1 and 2 μM) with Cr(VI) to BEAS-2B cells and measured the miR-21 level and PDCD4 expression at two, four and six months. We found that there was a significant (p<0.05) increase in the miR-21 level (Figure 4E) associated with a drastic decrease in the PDCD4 expression (Figure 4F) by chronic Cr(VI) exposure. Interestingly, co-treatment of quercetin markedly suppressed the Cr(VI)-induced miR-21 elevation and PDCD4 reduction in a time and dose dependent manner. Similar result was observed by immunofluorescence, where the co-treatment of quercetin prominently inhibited the Cr(VI)-induced suppression of PDCD4 expression in BEAS-2B cells (Figure 4G). These observations clearly demonstrate the chemopreventive effect of quercetin on Cr(VI)-induced oncogenic signaling and malignant transformation.

### Stable knockdown of miR-21 in BEAS-2B cells significantly reduces the Cr(VI)-induced cell transformation

**Figure 5:**
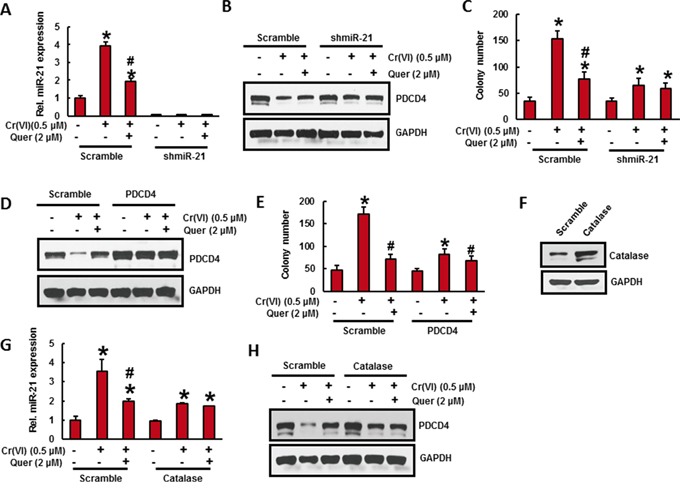
Stable knockdown of miR-21 and overexpression of PDCD4 or catalase in BEAS-2B cells significantly reduces the Cr(VI)-induced cell transformation Stable knockdown of miR-21 in BEAS-2B cells suppresses the Cr(VI)-induced cell transformation. **A-C.** BEAS-2B cells were stably knockdown with miR-21 shRNA or their corresponding vehicle vector and treated with Cr(VI) (0 or 0.5 μM) with or without quercetin (2 μM) for 6 months. (A) The relative miR-21 level was determined by Taqman real-time PCR. (B) Cell lysates were prepared to determine the protein level of PDCD4 using Western blot analysis. (C) Malignant cell transformation was determined by soft agar assay. Stable overexpression of PDCD4 in BEAS-2B cells reduces the Cr(VI)-induced cell transformation. **D-E.** BEAS-2B cells were stably overexpressed with PDCD4 or their corresponding vehicle vector and treated with Cr(VI)(0 or 0.5 μM) with or without quercetin (2 μM) for 6 months. (D) Cell lysates were prepared to determine the protein level of PDCD4 using Western blot analysis. (E) Anchorage independent growth was determined by soft agar assay. Stable overexpression of catalase in BEAS-2B cells decreases the Cr(VI)-induced cell transformation. **F-H.** BEAS-2B cells were stably overexpressed with catalase or their corresponding vehicle vector treated with Cr(VI) (0 or 0.5 μM) with or without quercetin (2 μM) for 6 months. (F) BEAS-2B cells overexpressed with catalase was determined by Wester blotting. (G) The relative miR-21 level was determined by Taqman real-time PCR. (H) PDCD4 protein level was detected by immunoblotting. Data presented in the bar graphs are the mean ± SD of three independent experiments. *# indicates a statistically significant difference from respective control cells with p<0.05.

To confirm the oncogenic role of miR-21 during Cr(VI)-induced malignant cell transformation, we stably knock down the miR-21 in BEAS-2B cells and treated with Cr(VI) (0.5 μM) for six months. As shown in Figure 5A, miR-21 stable knock down completely inhibited the miR-21 elevation even after chronic Cr(VI) treatment. Moreover, miR-21 shut down in BEAS-2B cells also inhibited the chronic Cr(VI)-induced suppression of its target tumor suppressor, PDCD4 (Figure 5B). Importantly, miR-21 knock down significantly suppressed the chronic Cr(VI)-induced malignant cell transformation (Figure 5C). The co-treatment of quercetin with Cr(VI) in miR-21 knockdown BEAS-2B cells have not exhibited any further significant changes in miR-21 level, PDCD4 expression, and colony number compared to miR-21 knockdown cells treated with Cr(VI) alone. These results demonstrated that elevation of miR-21 is important for Cr(VI)-induced malignant cell transformation.

### Stable overexpression of PDCD4 in BEAS-2B cells significantly reduces the Cr(VI)-induced cell transformation

To verify the functional role of PDCD4 in malignant cell transformation, we stably overexpressed the PDCD4 in BEAS-2B cell and chronically exposed to Cr(VI) (0.5) for six months. Western blot analysis revealed that forced expression of PDCD4 markedly suppressed the Cr(VI)-induced reduction of PDCD4 protein expression in BEAS-2B cells (Figure 5D). In addition, chronic Cr(VI)-induced malignant transformation (Figure 5E) was also significantly (p<0.05) decreased in PDCD4 overexpressed BEAS-2B cells. However, co-treatment of quercetin with Cr(VI) have not shown any further changes in PDCD4 expressionand colony formation compared to PDCD overexpressed cells treated with Cr(VI) alone.

### Catalase inhibits chronic Cr(VI)-induced miR-21 elevation and PDCD4 suppression

To study the preventive role of antioxidant on chronic Cr(VI)-induced miR-21 elevation and PDCD4 suppression, we overexpressed catalase in BEAS-2B cells (Figure 5F) and treated with Cr(VI) (0.5 μM) for six months. Stable overexpression of catalase remarkably inhibited the chronic Cr(VI)-induced miR-21 elevation (Figure 5G) and PDCD4 reduction (Figure 5H) in BEAS-2B cells, indicating that ROS plays a key role in chronic Cr(VI)-induced miR-21-PDCD4 signaling pathways. The co-treatment of quercetin have not shown any significant changes in miR-21 level and PDCD4 expression after Cr(VI) treatment in catalase overexpressed BEAS-2B cells.

### Quercetin inhibits the growth of xenograft tumors in mice from cells chronically exposed to Cr(VI)

**Figure 6:**
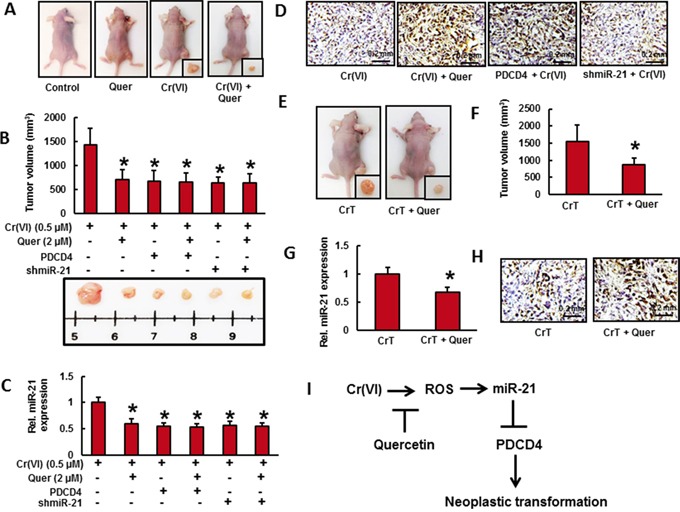
Quercetin inhibits the growth of xenograft tumors in mice from cells chronically exposed to Cr(VI) Cells from different treatments were injected into the flanks of 6-week old athymic nude mice (2×10^6^ cells per mouse). Mice were checked daily for tumor appearance, and tumor volume was measured after 30 days. Tumor volume was determined by Vernier caliper, following the formula of *A×B^2^×0.52*, where A is the longest diameter of tumor and B is the shortest diameter. **A.** Mice injected with BEAS-2B cells exposed to quercetin (2 μM) along with Cr(VI) (0.5 μM) showed reduced tumor incidence. **B.** Stable knockdown of miR-21 and overexpression of PDCD4 reduces the tumorogenicity of chronic Cr(VI) exposed BEAS-2B cells in nude mice. **C.** The relative miR-21 level was determined by Taqman real-time PCR. **D.** PDCD4 protein expression was detected by immunohistochemistry. **E.** CrT cells (2×10^6^ per mouse) were injected into the 6-week-old female athymic nude mice. After the tumors had developed (about 100 mm^3^), the mice were injected with or without 10 mg/Kg/day quercetin (ip) every day for 30 days. **F.** Solid tumors in the quercetin treated mice were significantly smaller than those in the control mice. Consistent with tumor volume data, tumors from quercetin treated animals showed a decreased **G.** miR-21 level and more **H.** PDCD4 positive cells compared to tumors from control mice. Data presented in the bar graphs are the mean ± SD of three independent experiments. * indicates a statistically significant difference from respective control with p<0.05. **I.** Proposed mechanism of quercetin inhibits Cr(VI)-induced malignant cell transformation.

To verify the chemopreventive effect of quercetin, nude mice were injected sc with BEAS-2B cells exposed to indicated concentration of Cr(VI) with or without quercetin for 6 months as shown in Figure 6A. We observed visible tumor formation that progressively increased in size in mice injected with Cr(VI)-treated BEAS-2B cells but not in those injected with untreated control cells after a period of 4-week post injection (Figure 6A). However, the mice injected with BEAS-2B cells exposed to quercetin along with Cr(VI) showed reduced tumor incidence (Figure 6A-6B). In addition we also demonstrated that stable knockdown of miR-21 and overexpression of PDCD4 reduces the tumorogenicity of chronic Cr(VI) exposed BEAS-2B cells in nude mice (Figure 6B). Consistent with our *in vitro* findings above, we found a significantly increased miR-21 level (Figure 6C) associated with decreased PDCD4 expression (Figure 6D) in xenograft tumors generated with chronic Cr(VI) exposed BEAS-2B cells. Consistent with tumor volume data, relatively less miR-21 level and more PDCD4 expression were observed in xenograft tumors generated with BEAS-2B cells chronically co-treated with quercetin and Cr(VI). Similarly, xenograft tumors generated with Cr(VI)-exposed BEAS-2B cells stably knockdown with miR-21 and overexpressed with PDCD4 were also showed relatively less miR-21 level (Figure 6C) and more PDCD4 expression (Figure 6D) in qRT-PCR and immunohistochemical analysis, respectively.

To further investigate whether quercetin inhibits tumor growth *in vivo*, CrT cells (2×10^6^ per mouse) were injected into the 6-week-old female athymic nude mice. After the tumors had developed (about 100 mm^3^), the mice were injected with or without 10 mg/Kg/day quercetin (ip) every day for 30 days (Figure 6E). We found that intraperitoneal administration of quercetin significantly suppressed tumor volume (Figure 6F). In addition, tumors from quercetin treated animals showed a decreased miR-21 level (Figure 6G) compared to tumors from animals injected with CrT cells. We also observed a less number of PDCD4 positive cells (Figure 6H) in untreated control group whereas a large number in quercetin treated group. All these observations indicate the chemopreventive and therapeutic efficacy of quercetin *in vivo* that strongly support the above *in vitro* studies.

## DISCUSSION

Hexavalent Chromium [Cr(VI)] compounds are well-known carcinogen associated with a higher incidence of human lung cancer [39]. Environmental exposure to Cr(VI) could cause lung toxicity in the short term and carcinogenicity over the long term [40]. Cancer chemoprevention using dietary antioxidant is a promising strategy for preventing Cr(VI) carcinogenesis. Many studies have reported the use of flavonoids as effective natural inhibitor on cancer initiation and progression [29, 41–43]. Among flavanoids, quercetin is one of the most potent anti-oxidants, as demonstrated in many studies [44–47]. Various cellular as well as animal models have reported the chemopreventive effects of quercetin [48–51]. In our laboratory, antitumor efficacy of quercetin was already investigated in both prostate and leukemia models [34, 52]. Previous study has shown that co-treatment with epigallocatechin-3-gallate (EGCG), the major polyphenol present in green tea, could protect BEAS-2B cells from Cr(VI)-induced cell death [53].

The oncogenic potential of miR-21 has been extensively studied in a variety of cancers [11, 54–57]. In particular, miR-21 was found overexpressed in lung cancers [1, 58, 59], and predicts poor patient survival [60, 61]. The tumor suppressor gene PDCD4 has been validated as a miR-21 target in prostate cancer [62], glioblastoma [63], retinoblastoma [64], lung cancer [8], thyroid carcinoma [65], colorectal cancer [66], and renal cell carcinoma [67]. miR-21 binds to the 3′-UTR of tumor suppressor PDCD4 and suppresses its translation [4]. Therefore, miR-21 and PDCD4 were considered as potential targets for novel cancer prevention or anti-cancer therapies. In our study we found that quercetin markedly inhibited both acute and chronic Cr(VI)-induced miR-21 elevation and PDCD4 reduction in BEAS-2B cells. In addition, Cr(VI)-induced binding of miR-21 to the 3′-UTR of PDCD4 was decreased by the treatment of quercetin. Treatment of quercetin also prominently inhibited chronic Cr(VI)-induced malignant cell transformation of BEAS-2B cells. Besides, stable knockdown down of miR-21 and overexpression of PDCD4 in BEAS-2B cells significantly inhibited the chronic Cr(VI)-induced malignant transformation. These results strongly demonstrate that quercetin inhibits Cr(VI)-induced malignant transformation by targeting miR-21-PDCD4 signaling pathway.

It has been established that Cr(VI)-induced ROS is vital for malignant cell transformation [3]. Hydrogen peroxide (H_2_O_2_) has been implicated in the elevation of miR-21 level and suppression of PDCD4 expression in vascular smooth muscle cells [68]. It was also reported that miR-21 modulates ROS levels through targeting SOD3 and TNFα [69]. Recently, we have shown that Cr(VI) induces p47^phox^, one of the NOX subunits is the key source of Cr(VI)-induced ROS [3, 35]. Our results revealed that treatment with quercetin considerably attenuated acute Cr(VI)-induced ROS generation and NOX activation in BEAS-2B cells. In addition, catalase overexpression in BEAS-2B cells was also significantly inhibited Cr(VI)-induced miR-21 elevation, PDCD4 suppression and malignant cell transformation. These observations clearly demonstrated that quercetin remarkably inhibited ROS and thereby suppressed the chronic Cr(VI)-induced miR-21-PDCD4 signaling and malignant cell transformation.

To explore whether co-treatment of quercetin inhibits chronic Cr(VI)-induced xenograft growth of tumors in mice, BEAS-2B cells chronically exposed to Cr(VI) with or without quercetin were injected into nude mice and tumor growth was monitored. We observed a visible tumor formation that progressively increased in size in mice injected with Cr(VI)-treated BEAS-2B cells but a reduced tumor incidence in those injected with BEAS-2B cells exposed to quercetin along with Cr(VI). In addition we also found that intraperitoneal administration of quercetin significantly suppressed the tumor volume in nude mice injected with CrT cells. Moreover, a decreased miR-21 level associated with increased PDCD4 expression was observed in tumors of quercetin treated animals. Furthermore, we also confirmed that stable knockdown of miR-21 and overexpression of PDCD4 reduces the tumorigenicity of chronic Cr(VI) exposed BEAS-2B cells in nude mice. All these observations indicate the chemopreventive efficacy of quercetin *in vivo* that strongly support the above *in vitro* studies.

In summary, our findings show that quercetin inhibited Cr(VI)-induced miR-21 elevation and associated inhibition of PDCD4 expression in BEAS-2B cells. Quercetin also inhibited Cr(VI)-induced ROS generation and protected BEAS-2B cells from malignant cell transformation. Nude mice injected with BEAS-2B cells chronically exposed to Cr(VI) in the presence of quercetin showed reduced tumor incidence compared to Cr(VI) alone treated group. Moreover, a decreased miR-21 level associated with increased PDCD4 expression was observed in tumors of quercetin treated animals. Furthermore, stable knockdown of miR-21 and overexpression of PDCD4 reduced the tumorogenicity of chronic Cr(VI) exposed BEAS-2B cells in nude mice. In short, quercetin protects BEAS-2B cells from Cr(VI)-induced malignant cell transformation by targeting miR-21-PDCD4 signaling pathway (Figure 6I).

## MATERIALS AND METHODS

### Antibodies and chemicals

Quercetin (>99% pure) was purchased from Sigma (St. Louis, MO, USA), dissolved in DMSO, aliquoted, and stored at −20°C. Potassium dichromate (K_2_Cr_2_O_7_) was purchased from Sigma-Aldrich (St Louis, MO). Dichlorodihydrofluoresceine acetate (DCFDA) was purchased from Molecular Probes (Eugene, OR). Antibody against PDCD4 was purchased from Cell Signaling Technology (Danvers, MA). Antibody against GAPDH was purchased from Santa Cruz Biotechnology, Inc. (Santa Cruz, CA).

### Cell lines and cell culture

BEAS-2B (Human bronchial epithelial cell line) was obtained from the American Type Culture Collection (Rockville, MD). Chromium transformed cells (CrT) were generated as described previously [35]. Cells were cultured in Dulbecco's modified Eagle's medium (DMEM) supplemented with 10% fetal bovine serum (FBS), 2mM L-glutamine, and 5% penicillin/streptomycin at 37°C in a humidified atmosphere with 5% CO_2_ in air.

K_2_Cr_2_O_7_ was used for Cr(VI) treatment. For short-term exposure of Cr(VI), cells were grown to 80–90% confluent, and then the medium was replaced with fresh DMEM medium containing 0.1% FBS for overnight before indicated Cr(VI) treatment. For chronic exposure of Cr(VI), the cells were continuously cultured in growth medium with indicated concentrations of Cr(VI).

### Plasmids and transfection

Plasmids DNA encoding human catalase was purchased from Origene (Rockville, MD). PDCD4 3′-UTR reporter plasmid was kindly provided by Dr. Yong Li (University of Louisville, USA). pcDNA3.1/PDCD4 plasmid was kindly provided by Dr. Hsin-Sheng Yang (University of Kentucky, USA). The overexpression of catalase or PDCD4 in BEAS-2B cells were performed using Lipofectamine™ 2000 (Invitrogen, Carlsbad, CA) according to the manufacturer's protocol. Briefly, BEAS-2B cells were seeded in 6-well culture plates and transfected with 4 μg plasmid at approximately 50% confluency. Cell clones resistant to G418 were isolated, overexpression of CAT and PDCD4 protein production were confirmed by immunoblotting.

The pLenti-III-miR-Off-has-miR-21-puro-GFP expression vector and the negative control vector pLenti-III-miR-Off- puro-GFP were purchased from Applied Biological Materials, Inc. (Richmond, CA). Human embryonic kidney 293T cells (ATCC, Manassas, VA) were transfected with lentiviral packaging vectors (ABM, Richmond, BC, CA) and lentiviral vectors expressing miR-Off-has-miR-21-puro-GFP or miR-Off- puro-GFP by Lipofectamine™2000 (Invitrogen, Carlsbad, CA) according to the manufacturer's protocol [36].

For generating stable miR-21 knockdown cell lines, two days after transfection, supernatants containing viral particles were harvested and used to infect BEAS-2B cells at approximately 70% confluence in DMEM supplemented with 8 μg/ml of polybrene using lentifectin reagent (ABM technologies) following the manufacture's protocol. 72 h after transfection, the medium was changed to the selection DMEM with 10% FBS and supplemented with 1μg/mL puromycin (sigma) to screen stable cell lines for further assay. Three weeks later, the cell clones were screened and further cultured in DMEM medium containing 1μg/mL puromycin and selected through FACS (Fluorescent Assisted Cell Sorting system).

### Cell viability assay

Cell viability was determined using 3-(4,5-dimethylthiazol-2yl-2,5-diphenyl tetrazolium bromide (MTT) assay. Active mitochondrial dehydrogenases in living cells metabolize MTT to a purple formazan dye, which is measured photometrically at 570 nm using a spectrophotometer as described previously [35].

### Intracellular ROS determination

H_2_O_2_ generation was examined using the fluorescent dye DCFDA as described previously [35]. The cells were cultured in 6-well plates (2×10^5^ cells/well) and treated with Cr(VI) (5 μM) for 6 h and incubated with DCFDA (10 μM) for 40 min at 37°C. Then, cells were trypsinized, washed twice with cold PBS, and analyzed by fluorescence-activated cell sorting (FACS Calibur, BD Biosciences). The fluorescence intensity of DCFDA was measured at an excitation wavelength of 492 nm and an emission wave length of 517 nm.

### Luciferase reporter assay

BEAS-2B cells transfected with the luciferase reporter constructs were seeded into 24-well plates (5 × 10^4^/well) and subjected to various treatments when cultures reached 80–90% confluence. Cellular lysates were subjected to a luciferase reporter assay (Promega, Madison, WI) using Glomax luminometer (Promega) as described previously [35]. The results are expressed as relative activity normalized to the luciferase activity in the control cells without treatment.

### Clonogenic assay

BEAS-2B cells (10^5^ cells) were seeded into each well of a 6-well plate and allowed to attach overnight. After the indicated exposure, cells were collected by trypsinization, three hundred cells were then reseeded into each of three dishes (60 mm diameter), and grown for 10 days. The cells were fixed with 2% formalin for 10 min and stained with 0.5% crystal violet stain and counted.

### NOX activity assay

NOX activity was measured by the lucigenin enhanced chemiluminescence method as described previously [3]. Briefly, cells were harvested and homogenized by sonication in cold lysis buffer (20 mM KH_2_PO_4_, pH 7.0, 1 mM EGTA, 1mM phenyl methyl sulfonyl fluoride, 10 μg/ml aprotinin, and 0.5 μg/ml leupeptin). Homogenates were centrifuged at 800 X g at 4°C for 10 min to remove the unbroken cells and debris, and aliquots were used immediately. To start the assay, 100-μl aliquots of homogenates were added to 900 μl of 50 mM phosphate buffer, pH 7.0, containing 1mM EGTA, 150 mM sucrose, 5 μM lucigenin, and 100 μM NADPH. Photon emission in terms of relative light units was measured in a Glomax luminometer (Promega) every 30 s for 5 min. There was no measurable activity in the absence of NADPH. Superoxide anion production was expressed as relative chemiluminescence (light) units (RLU)/mg protein.

### Anchorage-independent colony growth assay for Cr(VI)-induced cell transformation

Soft agar colony formation assay was performed as described previously [35]. BEAS-2B cells or BEAS-2B cells with stable overexpression of catalase or PDCD4 or BEAS-2B cells with stable knockdown of miR-21 were treated with 0.5 μM Cr(VI). The fresh medium was added for every 3 days. After 24 weeks, 1 × 10^4^ cells were suspended in 3 mL culture medium containing 0.35% agar and seeded into 6-well plates with 0.5% agar base layer, and maintained in an incubator for 4 weeks. The colonies greater than 0.1 mm in diameter were scored by microscopic examination.

The Cr(VI)-transformed cells from anchorage-independent colonies were picked up and continued to grow in DMEM. Passage-matched cells without Cr(VI) treatment were used as control.

### Quantitative real-time polymerase chain reaction (qRT-PCR)

Total RNA was extracted using Trizol (Invitrogen), and cDNA was synthesized by using TaqMan^®^ microRNA reverse transcriptase kit (Applied Biosystems, Foster City, CA, USA) as per manufacturer recommendations. Expression of miR-21-microRNA was determined by the TaqMan miRNA-assay (Applied Biosystems, Foster City, CA, USA), and normalized using the 2^-ΔΔCT -method relative to U6-snRNA. All TaqMan-PCRs were performed in triplicates run on Bio-Rad's MyiQTM single-color real-time PCR detection system.

### Western blot analyses

Cells lysates were prepared in ice-cold RIPA buffer (Sigma-Aldrich) with freshly added protease inhibitor cocktail. The lysate was then centrifuged at 12000 g for 10 min at 4°C and the supernatant (total cell lysate) was collected, aliquoted and stored at −80°C. The protein concentration was determined using Coomassie Protein Assay Reagent (Thermo, Rockford, IL). About 40 μg cellular proteins were separated using 6%–12% SDS-polyacrylamide gel and transferred to nitrocellulose membrane (Bio-Rad, Hercules, CA). Membranes were blocked with 5% fat-free dry milk in 1X Tris-buffered saline (TBS) and incubated with antibodies. Protein bands were detected by incubating with horseradish peroxidase-conjugated antibodies (Kirkegaard and Perry Laboratories, Gaithersburg, MD) and visualized with enhanced chemiluminescence reagent (Perkin Elmer, Boston, MA).

### Immunofluorescence analysis

BEAS-2B cells or BEAS-2B cellsexposed to Cr(VI) with or without quercetin were grown on coverslips in 6-well plates. The cells were fixed in 4 % paraformaldehyde followed by permeabilization with 0.2 % Triton X-100, blockage with 10 % horse serum in PBS solution, and incubation with PDCD4 (1:500) antibody in buffer A (1% BSA, 0.1% Triton X-100, 10% horse serum in PBS solution) for 1 h at 37°C. The cells were then incubated with Alexa Fluor 488 goat anti-rabbit secondary antibody and mounted using DAPI. The cells were visualized using digital confocal microscopy (Confocal Fluorescence Imaging Microscope, Leica TCS-SP5) or Olympus BX53 fluorescence microscope.

### Tumorigenesis studies

Athymic nude mice (NU/NU, Female, 6–8 weeks old; Charles River) were housed in a pathogen-free room in the animal facilities at the Chandler Medical Center, University of Kentucky. All animals were handled according to the Institutional Animal Care and Use (IACUC). Cells (2×10^6^ cells per mouse) from different treatments were re-suspended in serum-free medium with matrigel basement membrane matrix (BD Biosciences) at a 1:1 ratio (total volume=100 μl) and subcutaneously injected into the flanks of nude mice. Mice were checked daily for tumor appearance, and tumor volume was measured after 30 days. Tumor volume was determined by Vernier caliper, following the formula of *A×B^2^×0.52*, where A is the longest diameter of tumor and B is the shortest diameter. At the end of the experiment, mice were sacrificed and the tumors excised and snap frozen.

### Immunohistochemical staining

Five-μm thick frozen tumor sections were hydrated in phosphate buffered saline (PBS), and then non-specific binding sites were blocked with 10% horse serum in PBS and preceded according to Vectastain ABC Kit protocol (Vector Laboratories, Burlingame, CA). Briefly, the sections were incubated with rabbit anti-PDCD4 (1:100) antibody for 2 h at room temperature, washed and then incubated with biotinylated secondary antibody for 45 min followed by incubation with ABC reagent. After washing in PBS, sections were developed in DAB solution until the desired staining intensity was achieved. Finally, the sections were counterstained with hematoxylin.

### Statistical analysis

The values were presented as means ± SD. One-way analysis of variance (ANOVA) was used for statistical analysis. p<0.05 was considered significantly different.
